# Perceptual judgments made better by indirect interactions: Evidence from a joint localization task

**DOI:** 10.1371/journal.pone.0187428

**Published:** 2017-11-02

**Authors:** Pavel Valeryevich Voinov, Natalie Sebanz, Günther Knoblich

**Affiliations:** Department of Cognitive Science, Central European University, Budapest, Hungary; Tokyo Daigaku, JAPAN

## Abstract

Others’ perceptual judgments tend to have strong effects on our own, and can improve perceptual judgments when task partners engage in communication. The present study investigated whether individuals benefit from others’ perceptual judgments in indirect interactions, where outcomes of individual decisions can be observed in a shared environment. Participants located a target in a 2D projection of a 3D container either from two complementary viewpoints (Experiment 1), or from a single viewpoint (Experiment 2). Uncertainty about the target location was high on the front-back dimension and low on the left-right dimension. The results showed that pairs of participants benefitted from taking turns in providing judgments. When each member of the pair had access to one complementary perspective, the pair achieved the same level of accuracy as when the two individuals had access to both complimentary perspectives and better performance than when the two individuals had access to only one perspective. These findings demonstrate the important role of a shared environment for successful integration of perceptual information while highlighting limitations in assigning appropriate weights to others’ judgments.

## Introduction

When making perceptual judgments people often rely not only on their own perceptual abilities, but also on others’ perceptual abilities. Simply being exposed to others’ perceptual judgments can influence one’s own perceptual judgments [1–4]. Recent research has shown that people can be very effective in integrating perceptual information interpersonally when making judgements together if they have an opportunity to verbally communicate [5–7]. Less is known about how perceptual information is integrated interpersonally in indirect interactions, where individuals do not directly communicate but are exposed to evidence of one another’s judgments in a shared environment. The aim of the present study was to investigate to which extent observing one another’s perceptual judgments increases perceptual accuracy in a situation where two individuals have access to complementary or redundant visual information.

To illustrate, consider the following example. Imagine two people who intend to place a reading chair right under a spotlight mounted on the ceiling. Verbally exchanging their views on the correct location of the chair may help them improve the accuracy of their individual judgments. But what if one of them simply marks her preferred spot by moving the chair to this location, so that the other can then choose to rely on this information as she is trying to identify the correct location herself? Will it make a difference whether the two people provide their judgments from the same perspective, or from orthogonal positions in the room that differentially affect their ability to locate the chair relative to the spotlight on the depth dimension? Finally, will multiple iterations of providing and revising individual judgments increase their accuracy?.

Two different lines of research support the idea that individuals can benefit from being exposed to others’ judgments. First, research on group behavior has stressed the role of *indirect interactions*, a type of information exchange that relies on properties of the environment making outcomes of individual decisions persistent and observable [8]. Information exchange via indirect interactions (also referred to as *stigmergy*) has been proposed as an explanation for the benefits of collective decision-making in groups of animals that exhibit swarm features [9–11]. A key example of information exchange via indirect interactions is trail-laying in ants [12], where following pheromones or passively left hydrocarbon footprints [13], allows individual ants in some species to lock onto the better choice from two or more food sources [14, 15]. Likewise, humans may benefit from traces of others’ judgments, such as when a patch of downtrodden grass marks the best view from a hilltop.

Second, studies on social influence and studies on advice-taking have shown that individuals can benefit from combining their own and others’ judgments. Early research on conformity and social influence provided evidence that people shift their own judgments in the direction of others’ dissenting judgments [1, 4, 16–18]. According to Kameda and Tindale [19], such behavior can be adaptive from an evolutionary perspective. When individuals have imperfect knowledge, a positive effect of revising one’s judgment based on others’ judgments is canceling out the random error in individual judgments [20, 21]. More recent studies on advice taking [22] demonstrate that people generally benefit from having access to others’ judgments but rely too much on their own judgments [23]. When the judgment lies on a one-dimensional numeric continuum (e.g., judging the date of a historic event), people typically either average the two judgments, or select one of the two [21], but they rarely weigh the judgments according the expected quality of the ‘advice’. Other than early studies on social influence which often employed simple perceptual tasks [2, 17, 24], the literature on advice taking mainly used tasks that required judging abstract numerical information such as the dates of historical events [23] or estimates of product sales [25].

The aim of the present study was to investigate whether and how people benefit from one another’s judgments in a shared environment. We addressed three questions that follow from and extend the previous literature on advice taking and group decision-making trough indirect interactions. First, we asked whether a shared environment provides an effective medium for exchanging perceptual information. The main challenge for an individual is to weigh her own and another’s judgment appropriately. From a normative point of view, judgments from a more competent individual should be given larger weight [26]. To perform such a weighing a shared metric system is needed to evaluate and compare the quality of individual judgments. However, it is not trivial to establish such a shared metric system. One possible solution is to communicate certainty about one’s judgments, so that judgments people feel more certain about receive higher weight [5, 27]. Here we consider an alternative to verbal communication. It is possible that a shared environment is sufficient to provide a common reference and scale so that the accuracy of one’s own and others’ judgments can be directly derived from feedback provided in the shared environment. This predicts that people should adequately weigh their own and others’ judgments in shared environments even if they have no means of communicating how certain they feel about their judgments.

Our second question was whether and how people separately assess and weigh their own and another’s accuracy on multiple perceptual dimensions when forming and revising perceptual judgments in a shared environment. This ability would seem important for adapting judgments [19] to the multidimensional properties of natural environments [28–30]. As illustrated in the reading chair example above, if the task is to locate a target on a plane, one’s perception will be less accurate on the front-back dimension and more accurate on the left-right dimension. It has been demonstrated [31] that when people are simultaneously exposed to two sources of perceptual information, they integrate this information in a near-optimal fashion: They give higher weight to the source of information that is more reliable. However, it is an open question whether people can effectively integrate in a sequential manner their own and another’s judgments when they have access to complementary information. Therefore, we asked whether individuals selectively weigh others’ judgments depending on the quality of others’ access to different dimensions of perceptual information. As a baseline, we determined how well one individual can integrate complementary sources of perceptual information.

Third, we asked how two individuals mutually influence each other in a shared environment across multiple iterations of providing and refining judgments. This complements advice-taking experiments where advisors form their judgments in isolation (for an exception see [32]) and receive no feedback from the judge. According to Keil and Goldin [8], true interaction is characterized by reciprocal influence, that is, an estimate of a judge may causally influence subsequent information provided by an advisor. Using a task where two participants took turns in refining their respective individual judgments we tested the possibility that reciprocity in environment-mediated interactions can create a positive feedback loop [8, 10, 33] that continues to increase accuracy across consecutive interactions.

## Experiment 1

**Fig 1:**
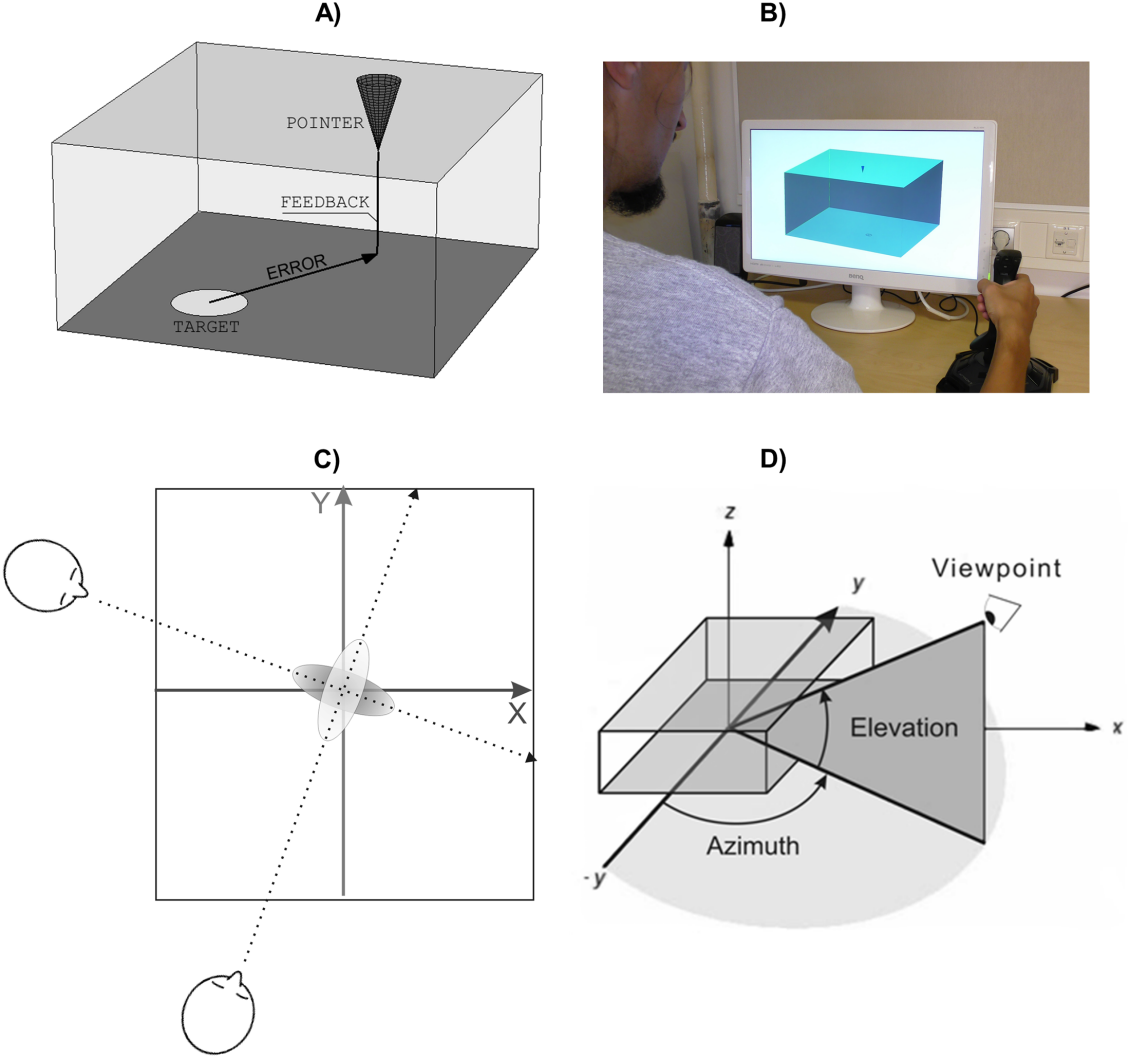
Experimental stimuli. (A) Schematic depiction of the localization task (the size of the pointer and the target were smaller in the actual task, see (B). Participants were asked to locate a POINTER above a target. FEEDBACK was provided once at the end of each trial by a line that projected the position of the pointer on the upper plane to the lower plane, visualizing the location error (ERROR). (B) Photograph of the experimental set-up in the IS condition. (C) Schematic representation of the top view in the Joint condition of Experiment 1 with a 90° difference in azimuth between viewpoints. Solid lines illustrate the intrinsic coordinates of the cuboid. Dashed lines illustrate participants’ egocentric front-back dimension for each of the two different viewpoints. The 90° difference in viewpoints implies that one participant’s accurate front-back dimension corresponded to the other participant’s accurate left-right dimension and vice versa. The two ellipses illustrate uncertainty about the target location for the two participants relative to the true target location. (D) Schematic illustration of parameters that define viewpoints on the cuboid in the virtual environment. Elevation (the angle relative to the *x*-*y* plane) was constant throughout the experiment. Azimuth (polar angle in the *x*-*y* plane) was varied to create different viewpoints.

To address these three questions, we developed a new localization task that was inspired by van Beers et al.’s work that addressed individuals’ weighing of information from different perceptual modalities in a localization task [31]. We asked pairs of participants to take turns in locating a target at the bottom of a 3D container displayed as a 2D projection on a monitor. Participants were asked to indicate the target position by placing a pointer on the top plane of the container exactly above the target (so that a perpendicular would connect its apex with the centre of the target, see Fig 1). Pilot studies confirmed that individual performance in this task corresponds to previous findings on visual localization [34]: judgment errors were scattered in elliptic distributions, elongated along participant’s virtual line of sight [35]. Participants’ accuracy was high on the left-right dimension (implying low variance of judgment errors) and low on the front-back dimension (implying high variance of judgment errors). This asymmetry reflects the lower accuracy of depth perception in the human visual system.

In an individual baseline (IS) participants judged the location of the target from a single viewpoint. In the joint condition (J) two participants took turns providing judgments from one particular viewpoint each (see Fig 1C). The angular difference between the two viewpoints was 90° so that the front-back dimension seen from one viewpoint corresponded to the left-right dimension seen from the other viewpoint and vice versa. Thus, together participants had complementary information that would have allowed them, in principle, to be very accurate.

The only information shared by two individuals in a pair was visual access to each other’s location judgment in a shared virtual environment. Each individual in a pair was instructed to be as accurate as possible, individually, to avoid any incentives for strategic cooperative or competitive behaviour. In a further “individual double” condition (ID) individuals switched between the two complementary viewpoints, which gave them the opportunity to observe their own judgment from an orthogonal viewpoint and to refine it accordingly.

If sharing the same (virtual) environment is sufficient to integrate interpersonally information about the target location, then judgments should be more accurate and less variable when individuals in a pair take turn with one another (J) than when the two individuals in the pair perform the task individually, from one particular viewpoint (IS). To the extent that others’ judgments are treated in the same way as own judgments, accuracy for pairs of individuals having complementary viewpoints (J) should approach the accuracy and variability of one individual switching between two complementary viewpoints (ID).

To the extent that participants weigh judgments according to each other’s estimated or perceived reliability separately for the two complementary perceptual dimensions, another’s individual’s judgement should be taken into account more when this individual has better perceptual information. Accordingly, another’s judgments on her left-her right dimension (corresponding to the participant’s front-back dimension) should have more impact on the participant’s judgment than another’s judgement on her front-back dimension (corresponding to the participant’s left-right dimension). Despite the large difference in individual accuracy between the front-back and right-left dimension (as a rule, accuracy differs by an order of magnitude), individuals who achieve optimal interpersonal integration should be able to achieve the same precision on the front-back dimension as on the right-left dimension. Finally, if taking turns in providing judgements (J) adds to the interaction anything above and beyond a one-time revision of one’s own judgment in the light of another’s judgment, judgments made after several iterations of interaction should be more accurate than those made after the first exposure to another’s judgment.

### Method

#### Participants

Thirty-two students (20 females, 12 males) aged between 19 and 26 years were tested in pairs and received payment for their participation. There were seven female, three male, and six mixed-gender pairs. One pair was replaced because of an experimenter error. The study was approved by the local ethics committee (Hungarian: Egyesített Pszichológiai Kutatásetikai Bizottság, EPKEB) and all participants gave written informed consent.

#### Material and apparatus

The task was presented using Apple iMac computers (2.5 GHz Intel Core i5 iMac computers with 21.4" Display and AMD Radeon HD 6750M 512 MB graphics) and an additional external monitor (BenQ RL2240H 21.5, connected to one of the Macs). The screen resolution on all monitors was 1600 X 900 pixels and all monitors were calibrated to have matched colour output. Location judgments were provided with Logitech Attack3 joysticks. The virtual environment for the location task was generated using the ‘perspective’ mode of the MatLab (The Mathworks, Inc.) software. The main element was a two-dimensional projection of a three-dimensional square-based rectangular cuboid (see Fig 1A) with a length × width × height ratio of 2×2×1. The cuboid was centred on the screen area (263×204 mm, expressed as dimensions of the MatLab axes rectangle) and simulated a real-world cuboid (256×256×128 mm). The target appeared in an inner area of the bottom plane of the cuboid with the constraint that it could not be closer to the edges than 1/5 of each side length.

The visible length of the sides of the cuboid on the screen varied as a function of the virtual camera orientation relative to the cuboid, according to the laws of perspective projection. Different viewpoints on the cuboid were generated by varying properties of the camera orientation as defined by azimuth (polar angle in the *x*-*y* plane) and elevation (angle above and below the *x-y* plane), see Fig 1D. Throughout the experiment, elevation was kept constant at 14°. The azimuth values were varied to generate different viewpoints on the layout. We used two orthogonal sets of viewpoints (90° difference in azimuth) with azimuth values of -70°/20° and -110°/-20°. The camera view angle was set to a constant 6.3° in an attempt to minimize angular distortions in the scene (the angle of an average telephoto lens ranges from 10° to 35°). The distance from the cuboid centre to the camera was constant.

To support tracking of the relations between different views on the cuboid, colour cues were used to highlight the cuboid orientation. The upper and lower planes of the cuboid were semi-transparent and coloured in teal. Its side planes were fully transparent. The edges defining one of the cuboid sides were coloured green and were 1.5 thicker than the other edges. These cues served to provide a stable percept of a cuboid in a particular orientation.

#### Procedure

Participants were instructed to locate a pointer in the upper plane of the cuboid precisely above a target placed on the lower plane of the cuboid, so that a perpendicular drawn from the pointer’s apex on the upper plane would connect the apex with the centre of the target on the lower plane (Fig 1A). The instruction highlighted that the location judgment should be as precise as possible. Participants were seated at an approximate distance of 60 cm from the screen throughout the experiment. There were two experimental conditions that involved complementary viewpoints. In the J condition two participants took turns in providing locations judgments from their respective viewpoints. The angular difference between viewpoints was kept constant at 90° throughout the experiment. In the ID condition, individual participants performed both parts of the task that was performed jointly in J, switching between the two complementary viewpoints. The IS condition served as a baseline for participants’ individual task performance from one particular viewpoint. All participants performed all conditions (J, ID, and IS).

The experimental session was divided into two blocks. Before the start of each block participants were familiarized with the two complementary viewpoints used in the ensuing block (4 trials). This ensured that participants were informed about the perceptual reliability associated with the two viewpoints and the complementary relation between the two viewpoints. Each block started and ended with 12 IS trials (IS-pre and IS-post) where individual participants provided location judgments from one viewpoint. The 12 trials in the middle of the block always involved judgments from two viewpoints, either by two different individuals (J) or by one individual (ID). The order of J and ID blocks and the viewpoint set used in each condition was counter-balanced across participants. There was a ten-minute break between the two blocks. In total, the duration of the experiment was up to 120 minutes.

In the J condition partners were seated in separate rooms looking at separate screens and operating separate joysticks. Before starting to work on the task they visited each other’s rooms and looked at each other’s setup. This served as a reminder about the complementary nature of the two viewpoints on the same virtual environment.

In each trial of the J condition participants took turn in providing judgements. They were instructed to be as precise as possible throughout (intermediate and final judgments). The order in which participants started the first trial was randomized. A sound signal indicated to the pair which partner would start. Each trial consisted of four judgment cycles comprising two turns each, so that both participants could adjust their initial judgments three times. In other words, a trial consisted of eight turns where one participant provided judgments on odd-numbered turns, and the other participant provided judgments on even-numbered turns. After the first trial the two participants alternated in providing either even or odd judgments.

Participants had 15 s to provide a judgment on each turn and a warning beep sounded after 10 s. Participants saw only the movements of their own pointer. The other’s pointer was visible at the location of her last judgement (or in the starting position on the starting participant’s first turn). When a participant’s pointer was active, it was shown in a specific colour assigned to the participant. When a participant’s pointer was dysfunctional it was coloured in grey. After the final judgment (fourth cycle, eighth turn), both participants received feedback about the location error of both pointers relative to the target at the position of their final judgment. Feedback consisted in a straight line projecting each pointer’s position on the upper plane to the lower target plane (see Fig 1A). No feedback was provided during intermediate turns (T1 through T7). An animated figure in S1 Fig provides an illustration of a joint trial procedure through the eyes of a participant.

The ID condition was the same as the J condition except that one participant switched back and forth between the two viewpoints for each of the four cycles. In the IS baseline individual participants located the target individually from a single viewpoint. Because there was only one viewpoint and no information reflecting another’s judgment was available, participants were not asked to revise their judgment as in the J and ID condition. The time limit for IS trials was 60 seconds to equate decision times with the ID and J condition.

#### Data preparation

Location judgments were coded as two-dimensional coordinates in the cuboid’s *x*-*y* plain. These coordinates were transformed into egocentric coordinates using the azimuth angle of the corresponding viewpoint. Accordingly, the resulting egocentric *Y* dimension was parallel to participants’ line of sight (front-back dimension), and the egocentric *X* dimension was parallel to the fronto-parallel plane (right-left dimension). The judgment error on each dimension was computed as the distance between the target coordinate and the judgment coordinate in egocentric coordinates. We excluded trials where the error on the front-back dimension exceeded three standard deviations of the front-back dimension error averaged across all conditions and participants the same criterion was applied. The trial was removed for both partners if one partner’s judgment exceeded the threshold. After this step 97.5% of the data were preserved for the ensuing analyses. From the uni-dimensional errors we computed absolute error, the Euclidean distance between the judgment coordinates and the true coordinates in mm (see Fig 1A). For the J condition we used each participant’s final judgment (fourth cycle) to compute judgment errors. For the ID condition judgment errors were computed as the average of the last two judgments (7^th^ and 8^th^ turn on the fourth cycle), one from each viewpoint. This is comparable to the J condition, because final judgment error in the J condition was derived from the 7^th^ and 8^th^ judgment as well: Each participant provided the last judgment on half of the trials and the penultimate judgment on the other half of the trials.

Because absolute error confounds systematic errors (bias) and variability of judgments, we also computed standard deviations for location error, as a measure of the reliability associated with location estimations [36]. In addition, we derived *impact weights* as a measure for the influence that observed judgments provided from another viewpoint exerted on the present judgment, separately for the front-back dimension and the left-right dimension (in analogy to percentage shift measured used by [37, 38]). Impact weight (or, shortly, *weight*) provides a direct normalized measure of how much a participant’s location judgments were adjusted towards judgments made from another viewpoint (provided by another person in the J condition and the participant herself in the ID condition. Weights were computed from trials where the participant provided judgments on odd turns (1, 3, 5, and 7) to ensure that the participant’s initial judgment was independent of observed judgments. Two types of weights were computed: serial and cumulative.

**Fig 2:**
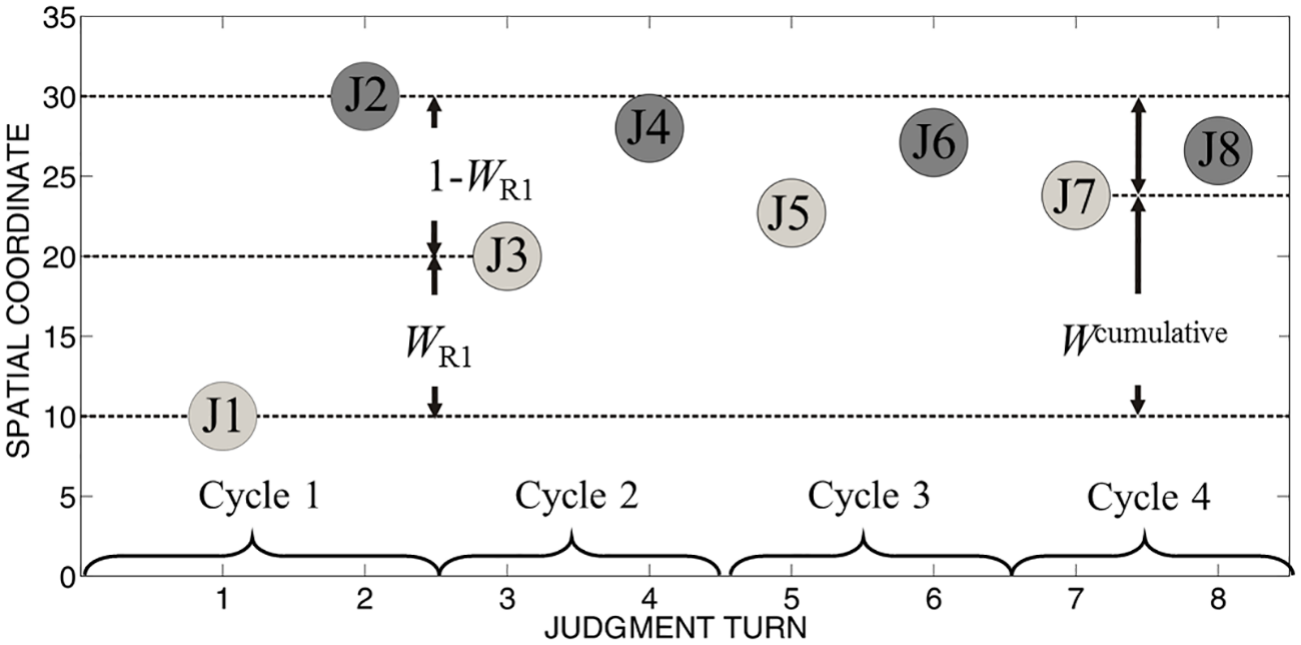
The course of a trial in Experiment 1 and illustration of impact weights. X-axis: In each trial 8 judgments from two different viewpoints were made. Y-axis: one-dimensional spatial coordinates in arbitrary units for illustration. In the J condition two participants took turn providing location judgments in four judgment cycles. In a given trial, participant 1 provided judgments on odd turns (1, 3, 5, and 7, in light grey) and participant 2 provided judgments on even turns (2, 4, 6, and 8, in dark grey). The impact of participant 2’s judgment on turn 2 on participant’s 1 judgment on turn 3 (i.e. the weight of participant 2’s judgment at the first iteration of judgment revision, *W*_R1_) was computed as the ratio of the spatial difference between participant 1’s judgment on turn 3 and participant 1’s judgment on turn 1 and the spatial difference between participant 2’s judgment 1 on turn 2 and participant 1’s judgment on turn 1. If a participant’s initial judgment on turn 1 was 10, the judgment from another viewpoint on turn 2 was 30, and the participant’s revised judgment on turn 3 was 20, then the impact of judgment 2 was 0.5 ((20–10)/(30–10)). The impact of another’s judgement 4 and 6 from a different viewpoint on judgment 5 and 7 was computed in the same way with judgment 3 and 5 as the respective base. The cumulative weight *W*^cumulative^ was computed as the ratio of the spatial distance between participant 1’s judgment on turn 7 and participant 1’s judgment on turn 1 and the spatial distance between participant 2’s judgment 1 on turn 2 and participant 1’s judgment on turn 1. If the final judgment of participant 1 was 24, the cumulative weight was 0.7((24–10)/(30–10)), reflecting additional influence exerted during reciprocal interactions.

Serial weights were computed as a measure of how much a participant’s immediate adjustments from judgment to judgment were influenced by observed judgments. These weights were computed separately for each adjustment (iteration 1 from J1 to J3, iteration 2 from J3 to J5, and iteration 3 from J5to J7), see Fig 2. To illustrate, we computed how an observed judgment on turn 2 (T2) impacted a participant’s judgment on turn 3 (T3), relative to the participant’s initial judgment on turn 1 (T1) as:

$$\frac{T3-T1}{T2-T1}$$

Cumulative weight was computed to determine whether the adjustments made during the course of interactions in a trial move the final judgment closer to the participant’s initially judged location from the same viewpoint as the final judgment or closer to the first observed location from another viewpoint. This can be expressed as

$$\frac{T7-T1}{T2-T1}$$

and provides a direct measure of the overall influence of initial location judgments from different viewpoints on the participant’s final location judgement.

### Results

The alpha-level for all statistical analyses was set to .05. We collapsed the data across viewpoint sets because there were no differences in location errors across different viewpoint sets. We first report the results of a pilot study that tested whether participants were indeed less accurate on the front-back dimension than on the left-right dimension when providing judgments from a single view point [35]. This is a crucial precondition for interpreting the results of Experiment 1.

**Fig 3:**
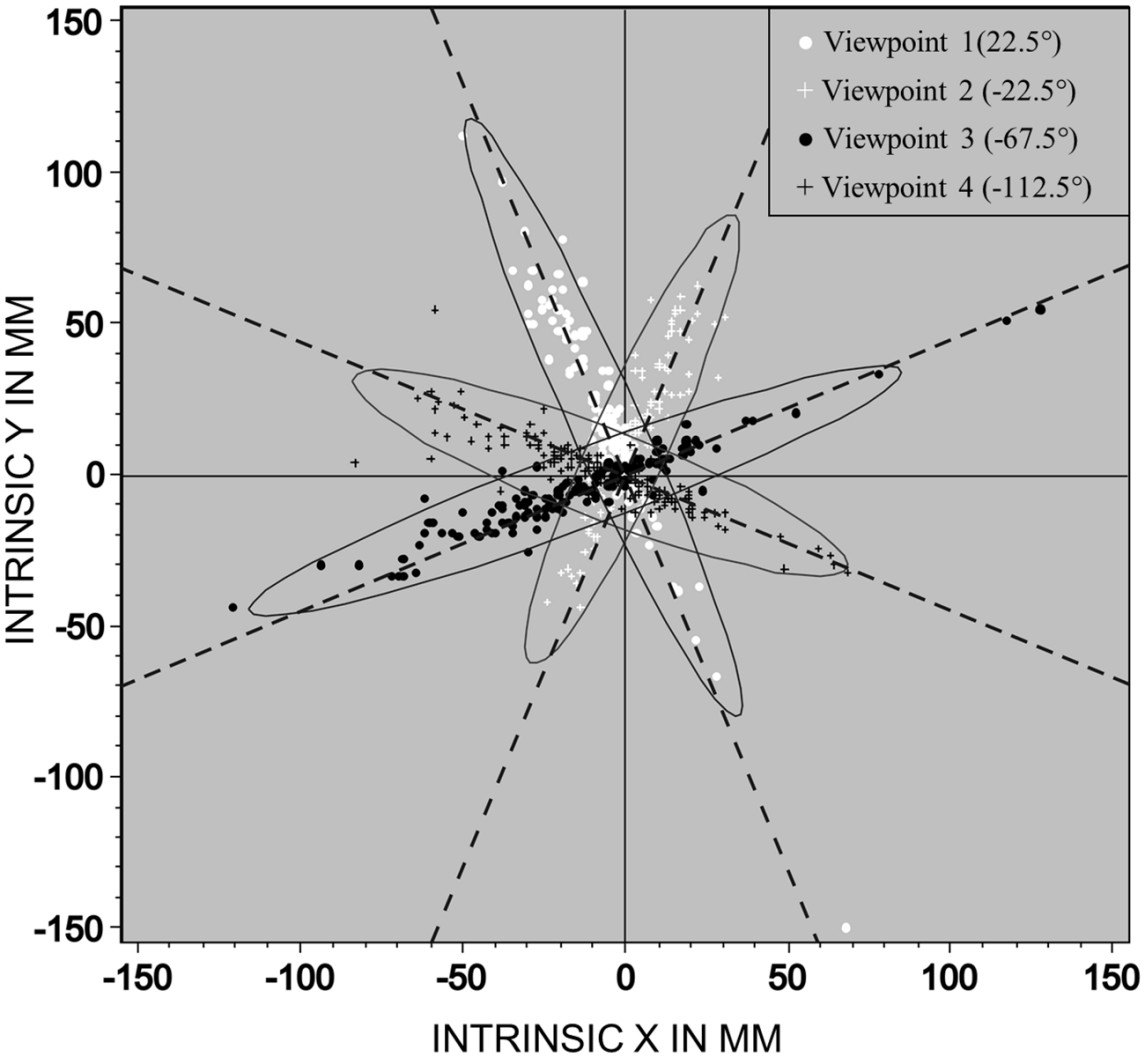
Raw distributions of errors in target location judgments. Distribution of individual location judgments from four different viewpoints. Coordinates are centered on the true target position so that deviations from 0, 0 reflect location error on the cuboid’s intrinsic spatial dimensions. The units are in millimeters. Ellipses are based on bivariate normal fits, where the perimeter of an ellipsoid corresponds to the 99% confidence curve. Participants’ variability was much higher on the egocentric front-back (major axes of the ellipses) dimension than on the left-right dimension (minor axes of the ellipses), as indicated by the elongation of the ellipses’ along the virtual lines of sight from the four different viewpoints (dashed lines).

Fig 3 displays raw judgment errors obtained in a pilot study where participants judged target locations from four different viewpoints that varied azimuth in four 45° degree steps. The figure illustrates that the different viewpoints led to the expected error distributions which could be well approximated with bivariate Gaussians generally meeting normality assumptions [35]. The error confidence ellipses were aligned with the respective viewpoints so that for each viewpoint the major axis of the ellipse was aligned with the virtual line of sight (dashed lines in Fig 3). Uncertainty in two egocentric dimensions was anisotropic: It was much higher on the egocentric front-back dimension (major axes of the ellipses) than on the egocentric left-right dimension (minor axes of the ellipses) resulting in an elongation of the ellipses. To test whether uncertainty on the front-back dimension was significantly larger than on the left-right dimension we compared the standard deviation of errors on these two dimensions. These were much larger on the front-back dimension, *t*(23) = 13.6, p < .001.

To test whether it is appropriate to analyse errors separately for the front-back dimension and the left-right dimension, we computed the Pearson correlation between the errors on these two dimensions separately for each viewpoint. None of the correlations were significant (all *p*s > .2). This justifies describing uncertainty about the target location in terms of two orthogonal egocentric dimensions.

#### Absolute error

**Fig 4:**
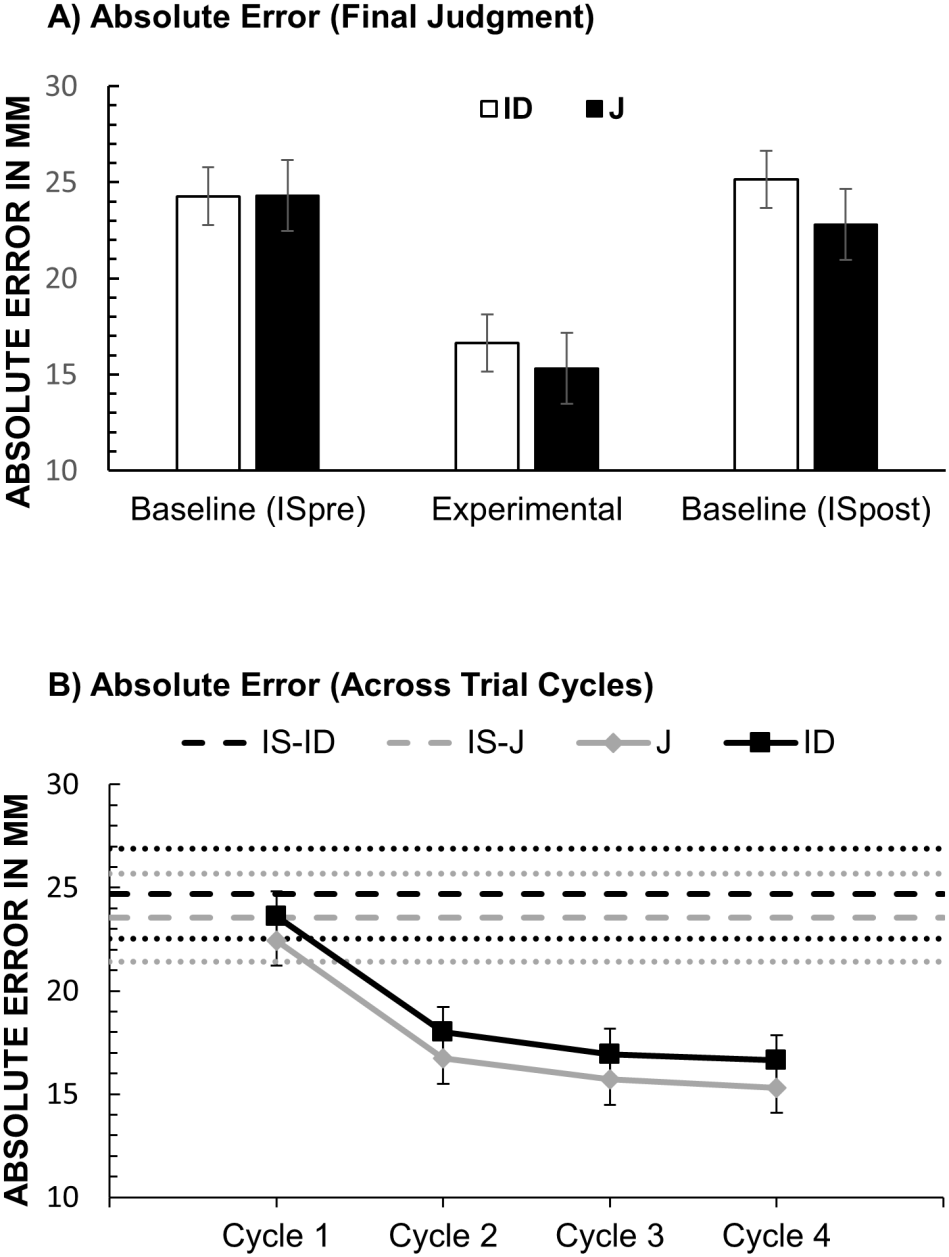
Results of Experiment 1: Absolute error. (A) Absolute error (mm) of final judgments (fourth cycle) in the ID and J condition and in the corresponding ISpre and ISpost baselines. Error bars stand for within-subject 95% CI [39]. (B) Absolute Error across consecutive cycles in the ID and J conditions. For each cycle error from two corresponding turns was averaged (e.g. Cycle 1 is the average of Turn 1 and 2). Error-bars stand for within-subject 95% CIs from the Condition × Cycle ANOVA (see main text). Dotted lines stand for the upper and lower SE of the means from the corresponding IS baseline (dashed lines).

Fig 4A shows the results for absolute error of location judgments in Experiment 1. To assess whether observing another’s judgment reduced participants’ location error we computed a one-way repeated-measures ANOVA with the factor Condition (IS-pre vs. J vs. IS-post). There was a significant effect of Condition, *F*(2, 62) = 13.9, *p* < .001, partial η^2^ = .310. Location error was lower in the J condition. To exclude the possibility that the lower error in the J condition reflected improvements due to learning we compared J to IS post in a planned contrast. Absolute error in the J condition (*M* = 15.3; *SD* = 15.5) was significantly lower than in the IS-post condition (*M* = 22.8; *SD* = 14.0 mm), *F*(1, 31) = 49.9, *p* < .001, partial η^2^ = .617. The difference between IS-pre and IS-post was not significant (*p* = .444).

The same ANOVA for the ID condition showed a significant effect, *F*(2, 62) = 20.2, *p* < .001, partial η^2^ = .395. A planned contrast revealed that Absolute Error in the ID condition (*M* = 16.6 mm; *SD* = 16.0 mm) was significantly lower than in the IS-post condition (*M* = 25.1 mm; *SD* = 13.6 mm), *F*(1, 31) = 36.6, *p* < .001, partial η^2^ = .542. The difference between IS-pre and IS-post was not significant (p = .474). A repeated-sample *t*-test showed no significant difference in absolute error between the ID and J condition, *p* = .552.

To assess whether overall accuracy improved across consecutive judgments we computed a 2 × 4 repeated-measures ANOVA with the factors Condition (ID vs. J) and Cycle (First vs. Second vs. Third vs. Fourth). There was a significant main effect of Cycle, *F*(3, 93) = 42.1, *p* < .001, partial η^2^ = .576. Planned contrasts showed that participants were more accurate on the second cycle (*M* = 17.4, *SD* = 14.9) than on the first cycle (*M* = 23.0, *SD* = 14.1), *F*(1, 31) = 47.4, *p* < .001, partial η^2^ = .604, and more accurate on the third cycle than on the second cycle (*M* = 16.3, *SD* = 15.3), *F*(1, 31) = 8.95, *p* = .005, partial η^2^ = .224. There was no significant difference between the fourth and the third cycle. The main effect of Condition and the interaction between Cycle and Condition were also not significant.

#### Variability of location judgments

**Fig 5:**
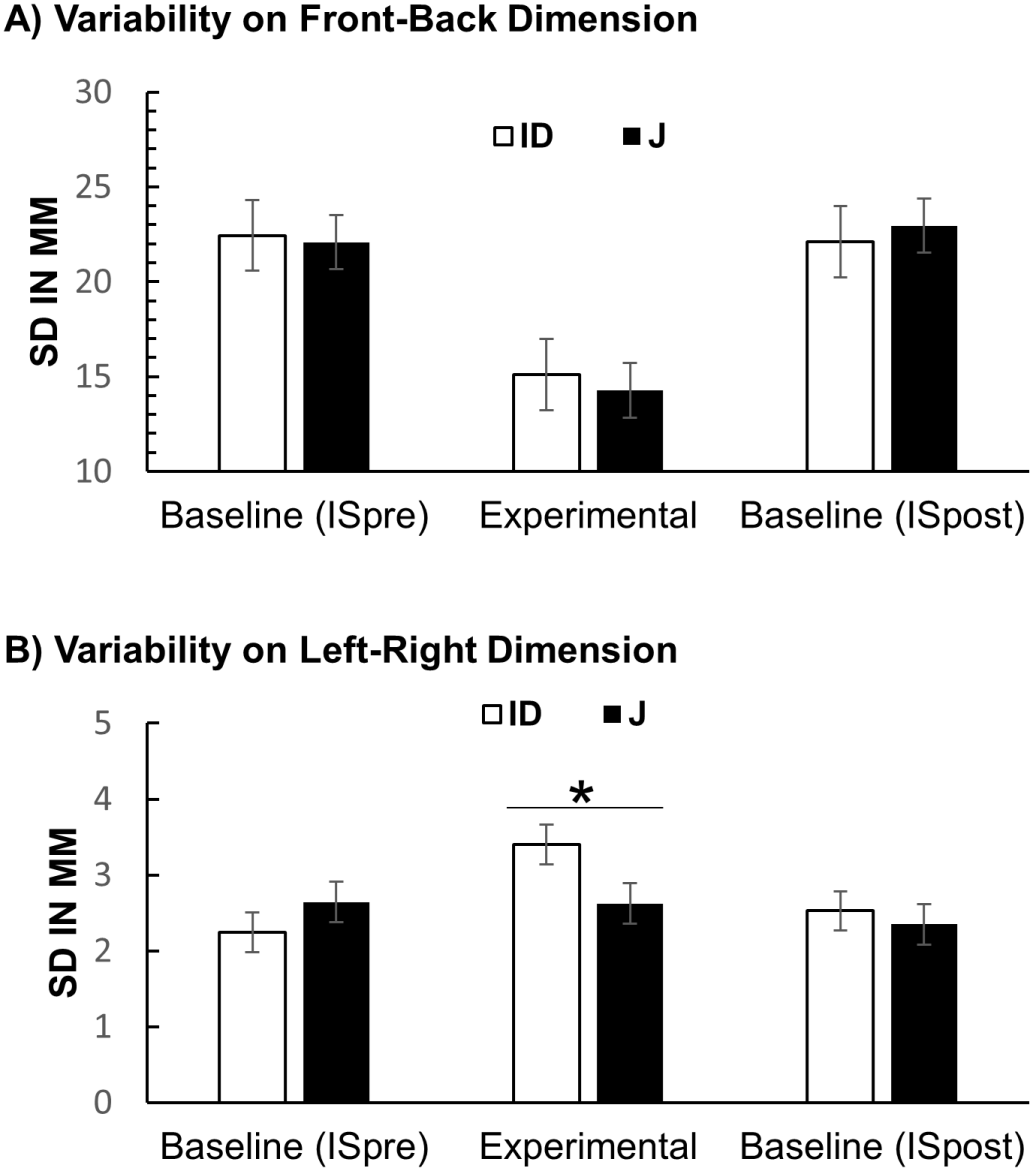
Results of Experiment 1: Variability of judgment errors. (A) Variability on egocentric front-back dimension. (B) Variability on egocentric left-right dimension. Error bars stand for within-subject 95% CI.

Fig 5A and 5B show the standard deviations of location judgment errors for the left-right and the front-back dimension, separately for the J condition and the ID condition. The standard deviations on the front-back dimension in the J condition were entered into a one-way repeated-measures ANOVA with the factor Condition (IS-pre vs. J vs. IS-post). For the front-back dimension there was a significant effect of Condition *F*(2, 62) = 22.9, *p* < .001, partial η^2^ = .425 (see Fig 5A) indicating that participants provided less variable judgments in the J condition. Planned contrasts showed that variability was significantly lower in the J condition (*M* = 14.3; *SD* = 9.24) than in the IS-post condition (*M* = 22.9; *SD* = 9.4), *F*(1, 31) = 48.8, *p* < .001, partial η^2^ = .611, ruling out the possibility that the lower variability in the J condition was due to learning. The difference between IS-pre and IS-post was not significant (*p* = .525). For the left-right dimension, there was no significant effect of Condition (see Fig 5B).

The same ANOVAs were conducted for the ID condition. For the front-back dimension there was a significant main effect, *F*(2, 62) = 10.1, *p* < .001, partial η^2^ = .246 (see Fig 4B). Planned contrasts revealed that variability in the ID condition (*M* = 15.1; *SD* = 12.6) was significantly lower than in the IS-post condition (*M* = 25.1; *SD* = 9.3), *F*(1, 31) = 14.6, *p* = .001, partial η^2^ = .320. The difference between IS-pre and IS-post was not significant (*p* = .805). The same ANOVA for the left-right dimension revealed a significant effect of Condition, *F*(2, 62) = 10.8, *p* < .001, partial η^2^ = .265. Unexpectedly, variability of participants’ judgments was higher in the ID condition than in the IS conditions.

Paired-samples *t*-tests were used to analyse whether standard deviations of location judgment errors differed between the ID and the J condition. For the front-back dimension the difference was not significant (*p* = .661). For the left-right dimension the difference was significant, *t*(31) = 2.112, *p* = .043. Standard deviations were smaller in the J condition (*M* = 2.62; *SD* = 1.76) than in the ID condition (*M* = 3.40; *SD* = 1.58).

#### Analysis of weights

**Fig 6:**
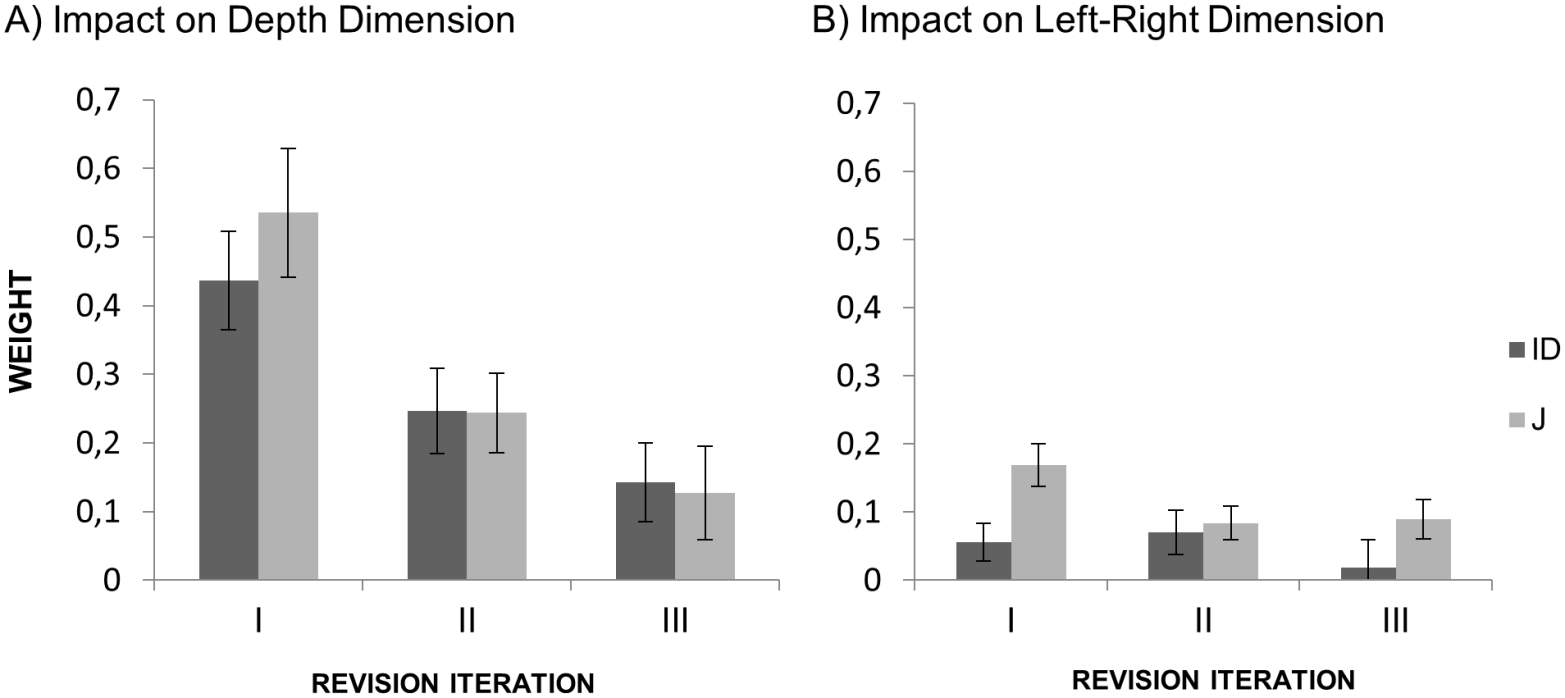
Impact of judgments from another viewpoint in Experiment 1. (A) Impact on front-back dimension. (B) Impact on left-right dimension. Error bars stand for within-subject 95% CI.

Serial weights were analyzed by means of a 2 × 3 repeated-measurement ANOVA with the factors Condition (J vs. ID) and Revision Iteration (R1 vs. R2 vs. R3). For the front-back dimension there was no main effect of Condition (*p* = .659) and no interaction between the two factors (*p* = .745). There was a significant main effect of Revision Iteration, *F*(2, 62) = 20.8, *p* < .001, partial η^2^ = .402, with weights decreasing across consecutive turns. The impact of the judgment 2, 4, and 6 was *M* = 0.49 (*SD* = 0.33), *M* = 0.24 (*SD* = 0.24), and *M* = 0.14 (*SD* = 0.24) respectively. Fig 6 illustrates the decrease of impact of judgments provided from another viewpoint across consecutive iterations.

For the left-right dimension there was a significant effect of Condition, *F*(1, 31) = 5.134, *p* < .001, partial η^2^ = .142. Serial weights were higher in the J condition (*M* = 0.11, *SD* = 0.11) than in the ID condition (*M* = 0.05 *SD* = 0.14). There was no significant effect of Iteration of Revision (*p* = .054) and no interaction between the two factors (*p* = .247).

Cumulative weights on the left-right dimension were smaller in the ID condition (*M* = 0.02, *SD* = 0.14) than in the J condition (*M* = 0.18, *SD* = 0.22). This indicates that in the J condition initial judgments originating from another participant had more influence on the participants’ final judgments than participants’ own initial judgments made from orthogonal viewpoint. For the front-back dimension the difference between the J and ID condition was not significant (*p* = .742).

### Discussion

The results of Experiment 1 support the prediction that a shared environment provides an effective medium for exchanging perceptual information. Two individuals taking turns in making location judgments from two different viewpoints (J condition) achieved the same level of absolute error as individuals switching between the same two viewpoints (ID condition). Furthermore, variability of errors in location judgments on the low-accuracy (front-back) dimension was reduced by the same amount in the ID and the J condition compared to the IS condition. Accordingly, indirect interactions where individuals observe each other’s judgments enable integration of perceptual information that reaches the same level of accuracy as judgments based on intrapersonal integration.

On the high-accuracy (left-right) dimension variability of judgment errors was significantly higher in the ID condition than in the J condition and in the IS condition. One potential explanation for this unexpected finding is that individuals switching between two perspectives in the ID condition were not able to maintain a viewpoint-neutral spatial representation [40]. This may have created a need for recalibration of viewpoints after each turn that was not required in the J condition.

The results also partially supported our prediction that the influence of observed judgments should be dimension-selective, and that it should be contingent on the reliability that can be achieved on each perceptual dimension. Accordingly, on the left-right dimension participants were weakly influenced by observed judgments: their revised judgments were nearly unchanged relative to their initial judgments. This was expected because on this dimension participants had an accurate perception of the target location, and the error in observed judgments could be clearly seen.

In contrast, on the inaccurate front-back dimension observed judgments had much higher impact on the revised judgments. However, participants’ revisions fell short of our expectation that revised judgments should be closer to the second (complementary) judgments than to initial judgments on the front-back dimension: this would be expected because judgments made from the orthogonal viewpoint provided a very reliable (in fact, nearly errorless) cue to the target location on this dimension. Surprisingly, accurate observed judgments influenced participants’ revised judgments only to the same extent as their own inaccurate initial judgments. This, however, was not specific to inter-individual interactions. Participants’ own judgments made from an orthogonal viewpoint were not weighed sufficiently in the same manner. This suggests that an anchoring process (well documented in the literature on judgment revision [41–43]) may have prevented participants from sufficiently relying on judgments made from an orthogonal viewpoint.

Our last question regarded the role of reciprocity in indirect interactions and how it affects individual performance. Our results clearly show that participants made more accurate location judgments in the second and third cycle of judgment exchange compared to the initial cycle. Our analyses of serial and cumulative weights provide further insights on how final judgments were iteratively formed in the course of indirect interactions. The impact of observed judgments progressively decreased with each iteration of judgment revision: participants were making progressively smaller adjustments relative to their most recent judgment. This made the revised judgments stabilize by the third (penultimate) cycle of judgment exchange (revised judgments were nearly unchanged after the third cycle).

Second, the analysis of cumulative weights revealed that on the low-accuracy (front-back) dimension participants’ final judgments tended to be closer to the observed judgments than to their own initial judgments: The cumulative weights in both J and ID condition approached the value of 0.7. This value is numerically larger than the average weights people give to external judgments in advice taking situations [22]. This result suggests that in the course of indirect interaction with multiple rounds of judgment and revision an individual judgment can be asymptotically attracted to a state where it is closer to another’s more accurate judgment than to the individual’s own initial judgment. The difference between the serial weights corresponding to the first iteration of judgment revision and the cumulative weights was not significant though. Thus, we cannot conclude that reciprocal interactions had a significant beneficial effect on the participants’ final judgments beyond the first revision.

## Experiment 2

Experiment 1 showed that indirect interaction can make two individuals with complementary viewpoints as accurate in their perceptual judgments as single individuals having access to both viewpoints. However, the results did not reveal any benefits of the joint condition that go beyond the accuracy individuals achieved in switching between two different viewpoints in the individual double condition. In contrast, prior research has shown that individuals who have the possibility to communicate certainty in their individual decisions about the same perceptual information can become more accurate in making joint judgments than the better individual in a pair [5, 6].

Therefore, our second experiment investigated whether exchanging location judgments by means of indirect interaction would lead to benefits beyond individual performance when two individuals provide judgments from the same viewpoint and thus have access to the same perceptual information. Even when individual judgments are based on the same shared visual input, they are prone to idiosyncratic (random) error inherent in individual perception. Converging towards a location in the present task could have a statistical effect of averaging, which is expected to filter out some random error in individual perceptual estimations [20, 21]. This should improve location accuracy in the J condition compared to the ID and the IS condition and lead to lower variability in judgment error. Alternatively, individuals could benefit from the mere opportunity to revise their judgments [44]. In this case the ID condition where participants had multiple opportunities to revise their judgments from one viewpoint should have the same benefits as the J condition compared to the IS condition.

### Method

#### Participants

Twenty-four paid students (20 females, 4 males) aged between 18 and 26 years (M = 21.5) were tested in same-gender pairs. One pair was replaced because of a program error, and one pair was replaced because of its poor performance on the task (3 SD worse than average in at least one experimental condition). The study was approved by the local ethics committee (Hungarian: Egyesített Pszichológiai Kutatásetikai Bizottság, EPKEB) and all participants gave written informed consent.

#### Stimuli and apparatus

The experimental stimuli and the set-up were identical to Experiment 1. However, we only used two different viewpoints with azimuth values of -110° and -70°.

#### Procedure and design

The procedure was identical to Experiment 1. In the J condition two participants were asked to make location judgments from the same viewpoint using one pointer each and in the ID condition one participant was asked to make two judgments from the same viewpoint using two different pointers.

### Results

#### Absolute error

**Fig 7:**
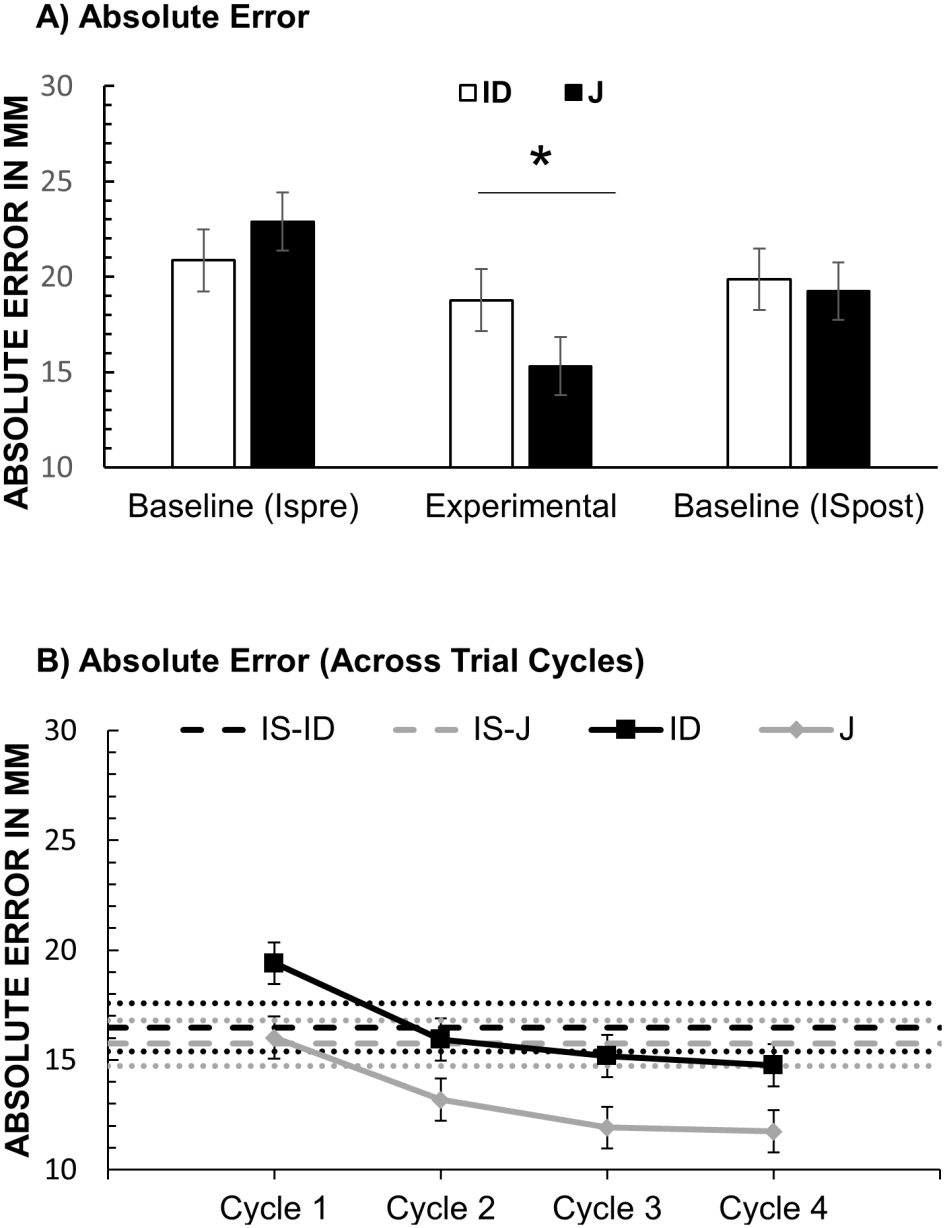
Results of Experiment 2. (A) Absolute error (mm) in the ID and J condition and in the corresponding ISpre and ISpost baselines. (B) Absolute error across consecutive trial cycles in the ID and J conditions. Error-bars stand for within-subject 95% CIs from the Condition × Cycle ANOVA (see main text).

Fig 7A shows the absolute error of location judgments in Experiment 2. A one-way repeated-measures ANOVA with the factor Condition (IS-pre vs. J vs. IS-post) showed a significant effect, *F*(2, 46) = 13.1, *p* < .001, partial η2 = .363. The contrast between the J condition (*M* = 11.7, *SD* = 3.88) and the IS-post (*M* = 13.34, *SD* = 5.85) condition was also significant, *F*(1, 23) = 4.80, *p* = .039, partial η2 = .173. These results demonstrate that two participants providing judgments from the same viewpoint were more accurate than one participant providing judgments from the same viewpoint.

The same analysis for the ID condition (IS-pre vs. ID vs. IS-post) also revealed a significant effect of Condition, *F*(2, 46) = 8.47, *p* < .001, partial η2 = .269. The contrast between the ID and IS-post was not significant (*p* > .5). A comparison of mean accuracy in the J and ID condition with a paired-sampled t-test showed that the absolute error in the J condition was significantly lower than in the ID condition (*M* = 14.85, *SD* = 5.72), *t*(23) = -2.62, *p* = .015.

To assess whether overall accuracy improved over consecutive judgments, we computed a 2 × 4 repeated-measurement ANOVA with the factors Condition (ID vs. J) and Cycle (First vs. Second vs. Third vs. Fourth). There was a significant main effect of Condition, *F*(1, 23) = 5.30, *p* = .031, partial η2 = .187 and Cycle, *F*(3, 69) = 16.3, *p* < .001, partial η2 =. 415. Absolute error was lower in the J condition (*M* = 13.2, *SD* = 4.21) than in the ID condition (*M* = 16.3, *SD* = 6.53). It was lower on the second cycle (*M* = 14.6, *SD* = 4.7) than on the first cycle (*M* = 17.7, *SD* = 6.32), *F*(1, 23) = 20.1, *p* < .001, partial η2 = .467, and lower on the third cycle (*M* = 13.56, *SD* = 4.17) than on the second cycle, *F*(1, 23) = 4.68, *p* = .041, partial η2 = .169. The difference between the third and the fourth cycle was not significant. There was no significant interaction between the two factors.

Fig 7B shows how absolute error changed over the trial cycles in the J and ID conditions. It can be seen that only in the J condition the absolute error eventually drops below IS baseline. This is different from Experiment 1, where absolute error dropped below the IS baseline in both J and ID conditions (see Fig 4B).

#### Variability of location judgments

**Fig 8:**
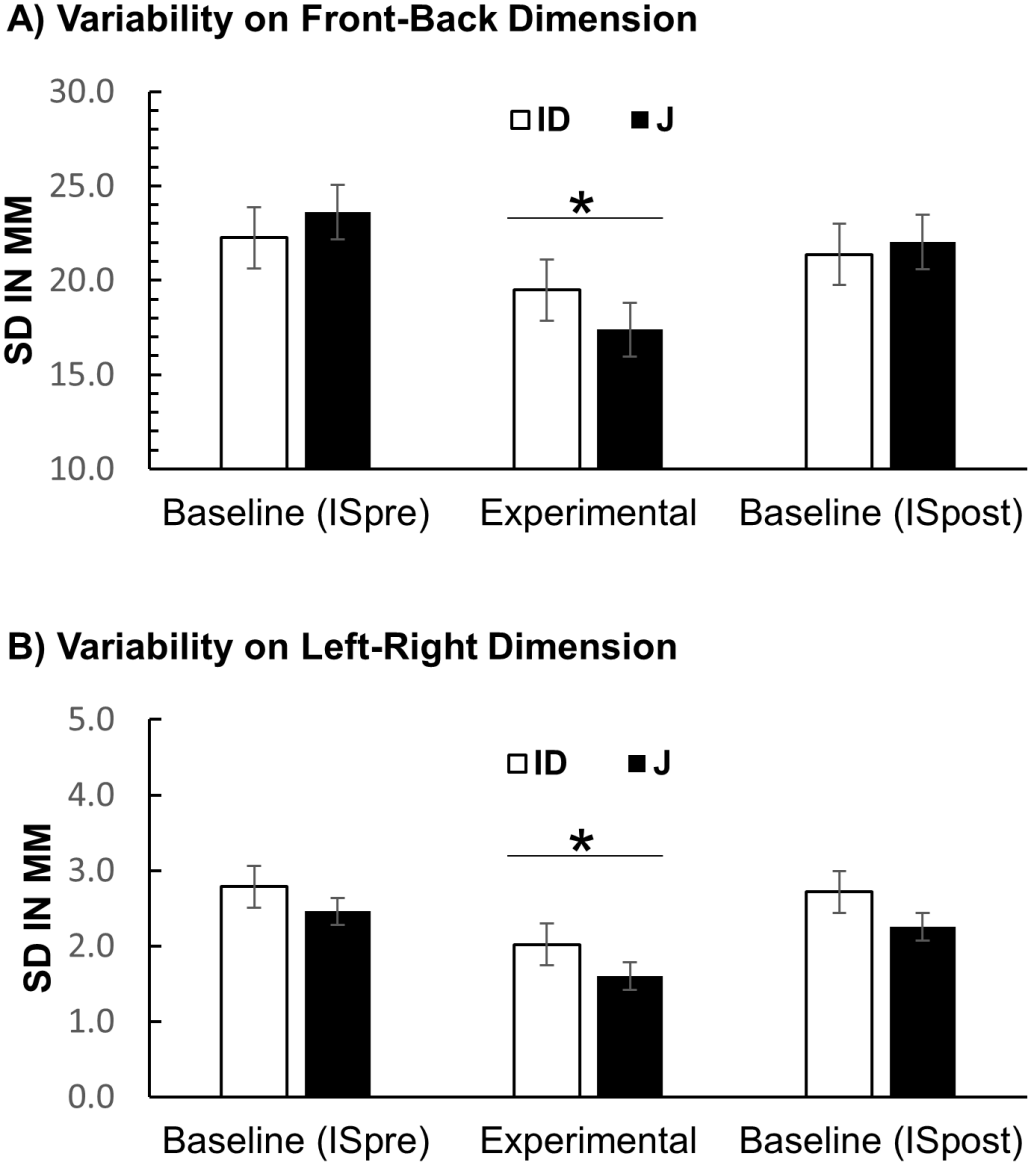
Results of Experiment 2: Variability of judgment errors. (A) Variability on egocentric front-back dimension. (B) Variability on egocentric left-right dimension. Error bars stand for within-subject 95% CI.

We first analysed variability of location judgments for the J condition. For the front-back dimension (Fig 8A) a one-way repeated-measures ANOVA with the factor Condition (IS-pre vs. J vs. IS-post) showed a significant effect of Condition, *F*(2, 46) = 14.0, *p* < .001, partial η2 = .394. However, the contrast between the J and the IS-post condition was not significant.

For the left-right dimension (Fig 8B) the effect of Condition was significant, *F*(2, 46) = 9.83, *p* < .001, partial η2 = .299. The difference between the J condition (*M* = 1.32, *SD* = 0.51) and the IS-post condition (*M* = 1.96, *SD* = 0.13) was significant, *F*(1, 23) = 9.92, *p* = .014, partial η2 = .234. The difference between the IS-pre and IS-post was not significant (*p* = .700). These results indicate that improvements on the left-right dimension were not simply due to practice with the task.

The same ANOVAs were performed for the ID Condition. There was no significant effect of Condition, neither for the front-back dimension nor for the left-right dimension (*p*s > .14).

Paired-samples *t*-tests were used to analyse whether standard deviations of location judgment errors differed between the ID and the J condition. Variability in the J condition was significantly lower than in the ID condition both on the front-back dimension, *t*(23) = -2.40, *p* = .025, and the left-right dimension, *t*(23) = -3.70, *p* = .001.

#### Analysis of weights

To analyze the impact of judgments repeatedly provided from the same viewpoint, 2 × 3 repeated-measurement ANOVAs with the factors Condition (J vs. ID) and Revision Iteration (First vs. Second vs. Third) were conducted. For the front-back dimension there was a significant main effect of Revision Iteration, *F*(2, 46) = 14.1, *p* < .001, partial η2 = .379. The impact of judgment 2, 4, and 6 was *M* = 0.54 (*SD* = 0.41), *M* = 0.15 (*SD* = 0.52), and *M* = 0.07 (*SD* = 0.14), respectively. There was no significant main effect of Condition (*p* = .329) or interaction (*p* = .156).

**Fig 9:**
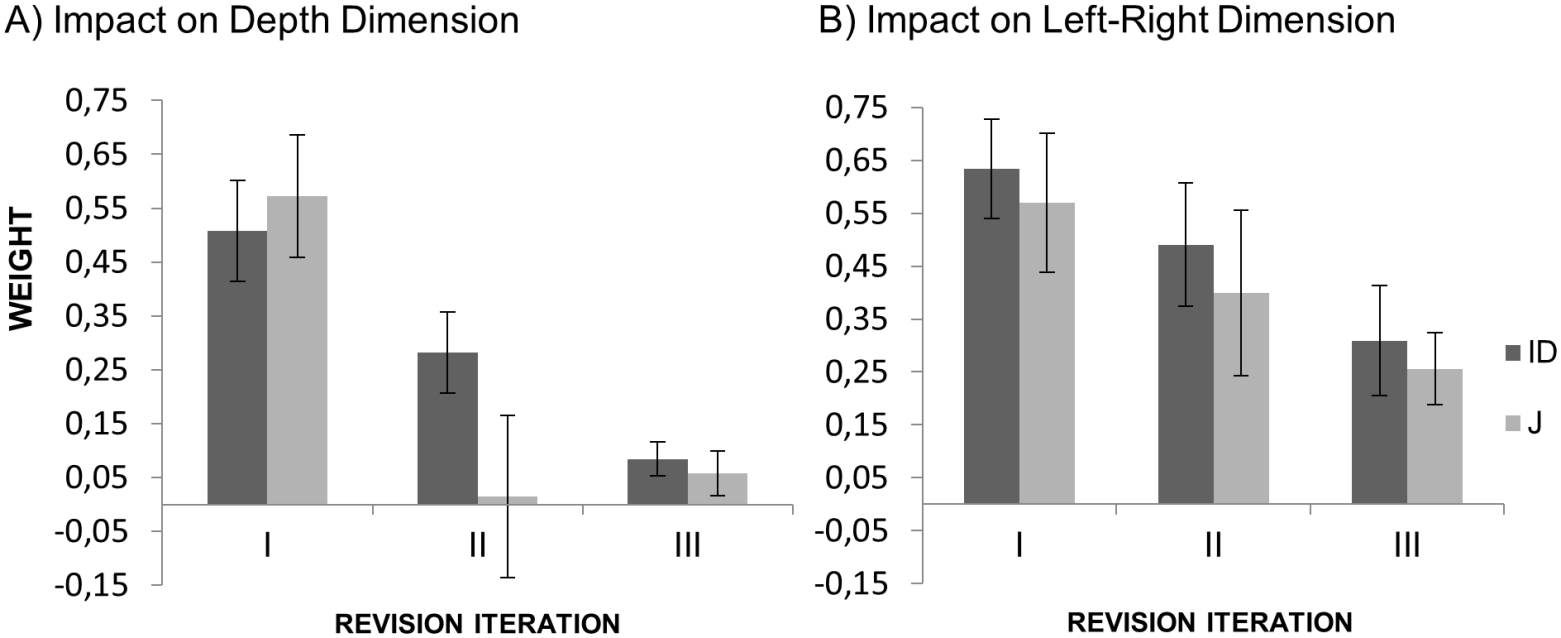
Impact of judgments from another viewpoint in Experiment 2. (A) Impact on front-back dimension. (B) Impact on left-right dimension.

The results were similar for the left-right dimension: There was a significant main effect of Revision Iteration *F*(2, 46) = 5.07, *p* = .010, partial η2 = .181. The impact of judgment 2, 4, and 6 was *M* = 0.60 (*SD* = 0.50), *M* = 0.44 (*SD* = 0.50), and *M* = 0.28 (*SD* = 0.35), respectively. There was no significant effect of Condition (*p* = .564), and no interaction (*p* = .98). Fig 9 illustrates that the impact of observed judgments decreased in both conditions and on both dimensions. This decreasing pattern of weights indicates that with each iteration of revision participants made progressively smaller adjustments to their most recent judgment.

Cumulative weights on the left-right dimension were generally lower in the J condition (*M* = 0.35, *SD* = 0.56) than in the ID condition (*M* = 0.63, *SD* = 0.55), *t*(23) = -2.32, *p* = .030. This means that participants’ own final judgments converged more closely to their own second judgments made from the same viewpoint than to another individual’s initial judgments.

To investigate whether participants applied different weighing strategies in Experiment 1 and Experiment 2, data from both experiments were analysed by means of a three-way 2 × 2 × 3 mixed ANOVA with the within-subject factors Condition (J vs. ID) and Revision Iteration (First vs. Second vs. Third), and the between-subject factor Viewpoint Difference (90° vs. 0°). Separate ANOVAs were run for the two dimensions. For the front-back dimension only the main effect of Revision Iteration was significant, *F*(2, 108) = 34.0, *p* < .001, partial η^2^ = .387. Serial weights at each iteration of revision were smaller than at the previous iteration. The impact of judgment 2, 4, and 6 was *M* = 0.51 (*SD* = 0.35), *M* = 0.20 (*SD* = 0.34), and *M* = 0.10 (*SD* = 0.20) respectively. Neither the main effect of viewpoint difference (*p* = .609) nor any interactions were significant.

For the left-right dimension the main effect of viewpoint difference was significant, *F*(1, 54) = 47.9, *p* < .001, partial η^2^ = .470. Serial weights were on average smaller in Experiment 1 (orthogonal viewpoints, *M* = 0.08, *SD* = 0.18) than in Experiment 2 (same viewpoint, *M* = 0.44, *SD* = 0.57). Similar to the front-back dimension, the main effect of Revision Iteration was also significant, *F*(2, 108) = 8.66, *p* < .001, partial η^2^ = .138. Serial weights at each iteration of revision were smaller than at the previous iteration. The impact of judgment 2, 4, and 6 was *M* = 0.32 (*SD* = 0.45), *M* = 0.23 (*SD* = 0.49), and *M* = 0.15 (*SD* = 0.34) respectively. There was also a significant interaction between Revision Iteration and Viewpoint Difference, *F*(2, 108) = 4.1, *p* = .019, partial η^2^ = .071. The decrease over consecutive iterations of revision was steeper in Experiment 2 (same viewpoint) than in Experiment 1 (orthogonal viewpoints), as can be seen by comparing Figs 6 and 9. This may be due to the serial weights on the left-right dimension being very low from the beginning in Experiment 1.

### Discussion

Experiment 2 supported the prediction that indirect interaction can benefit perceptual judgments when two individuals have the same viewpoint. The results showed that absolute error was lower when participants took turns in making location judgments (J) than when providing a single judgment (IS) or repeatedly revising their own judgments (ID). Furthermore, variability on the left-right dimension and the front-back dimension was lower in the J condition than in the ID condition. No benefits were present in the ID condition where one individual had twice the number of opportunities to revise her own judgments provided from one viewpoint. This result rules out the alternative explanation that the benefit of incorporating another’s judgments found in Experiments 1 and 2 can be attributed merely to the opportunity to revise one’s own judgments [44]. Instead, the results support the conclusion that the observed benefit comes from integrating judgments interpersonally.

The only deviation from our predictions was that variability on the more inaccurate front-back dimension was not significantly (albeit numerically) lower in J than in IS-post. This comparison is conservative because it assumes that the benefit of J outweighs general learning effects. The effect of Condition that included IS-pre and IS-post was highly significant. Fig 6B indicates that the improvement was not driven by a monotonic decrease in variability of error from IS-pre to IS-post which would be expected if it was only due to general learning. A comparison of the IS-post condition in Experiment 1 and 2 for the front-back dimension indicates that in Experiment 2 participants performed surprisingly well in the IS-post condition following the J condition. Thus, it is possible that in this experiment there were specific carry-over effects from the J condition that enabled participants to improve their performance in the IS-post condition.

As in Experiment 1, absolute error decreased across consecutive cycles and leveled out at the penultimate cycle both in the J and the ID condition. This decrease in error over revision cycles in the ID condition could be taken to imply that the accuracy increase over the consecutive cycles in the J condition may have been due to factors not related to social interaction (e.g. most participants were not fast enough to move the pointer to the intended location until the third cycle). Crucially, however, there was no indication that error or variability on either dimension could ultimately be reduced below the baseline (IS) level when individuals had the possibility to non-socially revise their own judgments (ID) making such influences very unlikely.

The analysis of cumulative and serial weights provided further insights into how participants’ judgments arrived at their final judgments. In line with our predictions, cumulative weights were close to the value of 0.5. The same was true for serial weights corresponding to the first iteration of judgment revision. This is a clear indication that when two individuals can assume comparable accuracy for their judgments, they tend to give another’s judgments nearly as much weight as their own.

An unexpected result was that on the more accurate left-right dimension participants’ own second judgment in the ID condition had significantly higher influence on their final judgment than another’s judgment in the J condition. A likely interpretation for this result is that a proportion of participants in the ID condition did not take very seriously their initial location judgments and only at the second turn started to provide location judgment that were close to the perceived target location. This could explain why their final judgment was closer to their second judgment than to their first judgment.

The comparison of Experiment 1 and Experiment 2 allows us to draw more general conclusions on how people are influenced by observable judgments under various conditions of uncertainty. The difference in how much participants were susceptible to observed judgments on their (accurate) left-right dimension confirmed the prediction that individuals are able to separately assess and weigh their own and another’s accuracy on multiple perceptual dimensions. For the left-right dimension, they were much less influenced by another’s judgments in Experiment 1, where another’s judgments on this dimension were substantially less reliable than the participants’ own judgments. When participants’ perceptual uncertainty was low they successfully discarded unreliable external information.

However, there was no difference between the two experiments in how much participants’ judgments were influenced by observable judgments on their inaccurate front-back dimension. Regardless of the reliability of another’s judgments, which was substantially higher on this dimension in Experiment 1, participants’ final judgments were not closer to the observed judgments from the other than to their own initial judgments. This suggests that when perceptual uncertainty was high, participants could not discriminate external information of higher and lower quality and were influenced by the two to the same extent.

## General discussion

In the current study, we investigated whether and how individuals can benefit from each other’s judgments by indirectly interacting in a shared environment. Performing a localization task, participants observed judgments made by another individual and were provided with the opportunity to take turns in revising their judgments upon exposure to the other’s most recent judgment. In Experiment 1 two individuals had complementary viewpoints on a shared environment and in Experiment 2 they had the same viewpoint on a shared environment.

Our aim was to investigate whether a shared environment provides an efficient medium for inter-individual integration of perceptual information. The results of both experiments showed that performing the task with another individual led to higher accuracy and lower variability of error in location judgments than performing the task alone from one viewpoint. Two individuals who were exposed to each other’s judgements from two complementary viewpoints reached the same level of accuracy as who switched between the two viewpoints. When providing judgments from the same viewpoint, two individuals looking from the same viewpoint were more accurate than when single individuals who had comparable opportunities to revise their individual judgments from the same viewpoint without observing another’s location judgments. Thus, the increase of accuracy in location judgments during indirect interactions resulted from inter-individual information integration, and was not simply due to better opportunities to revise one’s own judgments [44].

Our results extend earlier work on interpersonal integration of perceptual information [5, 6] by demonstrating that observing another’s perceptual judgments in a shared environment improves an observer’s judgments even in the absence of verbal communication. While previous research has emphasized the role of meta-cognition [6, 45] and suggested that communication may be a necessary precondition for individuals to benefit from each other’s judgments [6], the role of meta-cognition might be limited in simple behavioural decisions. Just as in the case of pheromones [12] much of the information relevant for interpersonal information integration could be implicitly and non-intentionally transmitted and perceived in a shared environment. It is an open question how the efficiency of indirect interaction and explicit communication for perceptual integration compare.

The second aim of our study was to investigate whether people can separately assess and compare their own and another’s accuracy on multiple perceptual dimensions. The results with respect to this question are mixed. Experiment 1 provided evidence that participants can apply a dimension-selective weighing strategy to another’s judgment, as they were more influenced by observed judgments on their inaccurate front-back dimension than on their more accurate left-right dimension. They discarded the front-back component of observed judgments because they had considerable better perception themselves on this dimension.

Whether people accurately assess the reliability of a particular dimension of another’s judgment and properly rely on it seems to depend on their own uncertainty about this dimension. On their inaccurate front-back dimension, participants weighed another’s judgments nearly as much as their own both when the judgments were approximately equally accurate (Experiment 2) and when another’s judgments were considerably more accurate (Experiment 1). In this way participants exhibited a remarkable lack of sensitivity to components of another’s judgment that were highly reliable.

This lack of sensitivity echoes recent findings [46, 47] indicating that human decision making is subject to an equality bias. This refers to a tendency to perceive another’s competence as being closer to one’s own competence than it actually is. In the present task such an equality bias existed only for the judgment component for which perceptual uncertainty was high. For the component for which perceptual certainty was low, participants radically discounted another’s judgments, just as people do when they receive inaccurate advice [23], or when they are very knowledgeable on the matter in question [48]. This aspect of our findings, taken together with those obtained by Yaniv and colleagues [23, 48], seems to support a hypothesis originally put forward by Deutsch and Gerard [17]: Higher uncertainty in one’s own judgment leads to higher susceptibility to social influence but not to an extent that makes them trust others’ judgments more than their own [17, p. 630].

Interestingly, individual participants also failed to optimize weighing information from two viewpoints. In the ID condition of Experiment 1, participants could have relied on their own previous judgment from another viewpoint to compensate for their inaccurate front-back judgments. However, variability of error on the front-back dimension was not lower than in the Joint condition. This similarity between the ID and the J condition indicates that intra- and inter-individual integration of information may have been compromised in the same manner and that a common cause prevented participants from relying enough on the most informative components of observed judgments.

This is not to say, however, that people cannot form distinct representations [49] of the front-back dimension and the left-right dimension. Experiment 1 clearly demonstrates that participants applied different weights on the two dimensions in the process of judgment revision. Their only problem was to give sufficient weight to accurate judgments they observed on the front-back dimension where they experienced high perceptual uncertainty. Interestingly, findings obtained in similar location tasks demonstrate that individuals do not have the same problems when they combine two simultaneously available sources of information [31], or when they continuously adjust a single judgment switching between complementary viewpoints [35]. Thus, the problems of not giving enough weight to useful information seems to be specific to judgments that are sequentially revised. This suggests that in inter-individual as well as in intra-individual information integration, evidence that is directly available dominates over evidence that is imagined or retrieved from memory. This explanation is in line with classical findings that there is consequent anchoring to immediate evidence in the process of judgment revision [43].

The third aim of our study was to investigate the role of reciprocity in the process of information integration during indirect interactions. Although we found that judgments improved in accuracy after several iterations of exchange and revision, we did not find evidence that reciprocal interactions led to a stronger improvement in the joint condition than in the condition where individuals switched between viewpoints. A general decrease in the influence of observed judgments over consecutive rounds of judgments exchange has been reported in previous studies on judgment revision [50] and shares similarities with the phenomenon of decreasing concessions observed in the process of bargaining on a compromise position in a situation of conflicting preferences [51]. One potential explanation for the decrease of influence in the present study is that participants felt greater confidence in their own judgments with each iteration but did not update their confidence in observed judgments in the same manner. Considering that tighter coordination and increased social influence can help to avoid biases in decision-making [6, 47], it would be interesting to explore in future studies whether the requirement for individuals to agree on a joint judgment makes them weight a task partner’s judgments in a more appropriate way.

## Supporting information

S1 Fig Joint trial sequence. A reconstruction of a representative joint trial though the eyes of a participant manipulating the red pointer. On the first frame of the animation the red pointer is already in the location submitted as the first (T1) judgment. The text box was not visible during the experiment.
(GIF)
