# Supplementary figures and images for: Perceptual judgments made better by indirect interactions: Evidence from a joint localization task
Source: PLoS One. 2017 Nov 2;12(11):e0187428. doi: 10.1371/journal.pone.0187428 (PMC5667753; doi:10.1371/journal.pone.0187428)

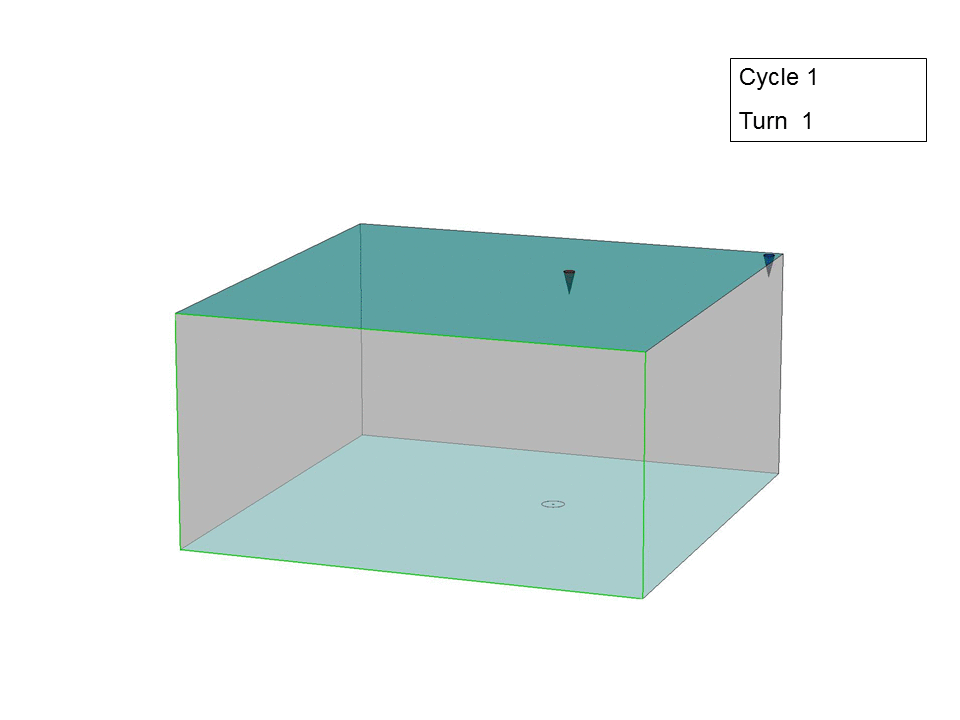

Supplement: S1 Fig — A reconstruction of a representative joint trial though the eyes of a participant manipulating the red pointer. On the first frame of the animation the red pointer is already in the location submitted as the first (T1) judgment. The text box was not visible during the experiment. (GIF) [file pone.0187428.s001.gif]
